# Can ^99^Tc^m^-3PRGD_2_(α_ν_β_3_) and ^18^F-FDG dual-tracer molecular imaging change the therapeutic strategy for progressive refractory differentiated thyroid cancer: Case report

**DOI:** 10.1097/MD.0000000000032751

**Published:** 2023-02-03

**Authors:** Yu Zhang, Yuxuan Li, Zhiyi Lin, Wenxin Chen

**Affiliations:** a Department of Nuclear Medicine, Shengli Clinical Medical College of Fujian Medical University, Fujian Provincial Hospital, Fuzhou, China; b Fujian Research Institute of Nuclear Medicine, Fuzhou, China; c Department of Nuclear Medicine, Shengli Clinical Medical College of Fujian Medical University, Fujian Provincial Jin Shan Hospital, Fuzhou, China.

**Keywords:** 18F, arginine-glycine-aspartic (RGD), differentiated thyroid cancer, fluorodeoxyglucose, PET/CT, tyrosine kinase inhibitors

## Abstract

The management of radioiodine refractory differentiated thyroid cancer (RAIR-DTC) represents a major challenge in thyroid cancer. The American Thyroid Association guidelines recommend the use of tyrosine kinase inhibitors (TKIs) for RAIR-DTC that does not respond to conventional treatment. Currently, imaging modalities that predict the response to TKI treatment based on morphological and functional features are lacking. we report a case of a patient with progressive RAIR lung metastases who underwent 2-deoxy-2-[^18^F]fluoro-D-glucose and 99technetiumm-three polyethylene glycol spacers-arginine-glycine-aspartic acid (^99^Tc^m^-3PRGD_2_) dual-tracer imaging and investigate the value of this imaging strategy for determining subsequent therapeutic schedules.

**Patient concerns:**

A 52-year-old man with advanced RAIR-DTC and progressive lung metastasis. After TKI treatment [sorafenib] lost its clinical benefits, the patient’s therapeutic response was evaluated as progressive disease. 2-deoxy-2-[^18^F]fluoro-D-glucose PET/CT and ^99^Tc^m^-3PRGD_2_ SPECT/CT were performed. There were multiple FDG-positive lesions in the lung. However, ^99^Tc^m^-3PRGD_2_ SPECT/CT showed only 1 lesion in the right middle pulmonary lobe with arginine-glycine-aspartic positivity.

**Diagnosis:**

RAIR-DTC.

**Interventions:**

Radiofrequency ablation was performed for only the lesion with RDG and FDG positivity.

**Outcomes:**

The patient quickly achieved partial response.

**Lessons:**

This case indicates that for progressive RAIR metastases, patients can benefit more from prioritizing treatment for lesions that are both arginine-glycine-aspartic and FDG positive.

## 1. Introduction

Thyroid cancer (TC) is one of the most common malignant tumors of the head and neck. Differentiated TC (DTC) originates from the thyroid follicular epithelium and accounts for more than 95% of TC cases.^[1]^ However, 1% to 23% of DTC patients develop distant metastases, of which 30% progress to radioactive iodine-refractory TC (RAIR-DTC).^[2,3]^ The 10-year survival rate of RAIR-DTC is less than 10%.^[4]^ Thus, theranostics for RAIR-DTC have become a clinical focus. In recent years, a variety of tyrosine kinase inhibitors (TKIs) have emerged and have been confirmed to prolong the progression-free survival of patients with RAIR-DTC.^[1,4]^ Ultrasound and computed tomography (CT) are commonly used in clinical trials to evaluate treatment response according to the Response Evaluation Criteria in Solid Tumors, relying on changes in tumor size.^[5]^ However, changes in metabolic function often precede changes in morphology. Anatomic changes often fail to evaluate the response to treatment in a timely manner, particularly for TKI therapy. Therefore, we report the case of a patient with progressive RAIR lung metastases who underwent 2-deoxy-2-[^18^F]fluoro-D-glucose (^18^F-FDG) and 99technetiumm-three polyethylene glycol spacers-arginine-glycine-aspartic acid (^99^Tc^m^-3PRGD_2_) dual-tracer imaging and investigate the value of this imaging strategy for determining subsequent therapeutic schedules. Our case report encourages further exploration of ^18^F-FDG and ^99^Tc^m^-3PRGD_2_ dual-tracer imaging for evaluating the response to TKI treatment in a timely manner.

## 2. Patient information

A 52-year-old man with RAIR-DTC and progressive lung metastasis received oral sorafenib 0.4 g twice daily. He had been smoking for about 40 years and drinking alcohol for about 20 years. The rest of his past medical history was unremarkable.

## 3. Clinical findings

**Figure 1.**
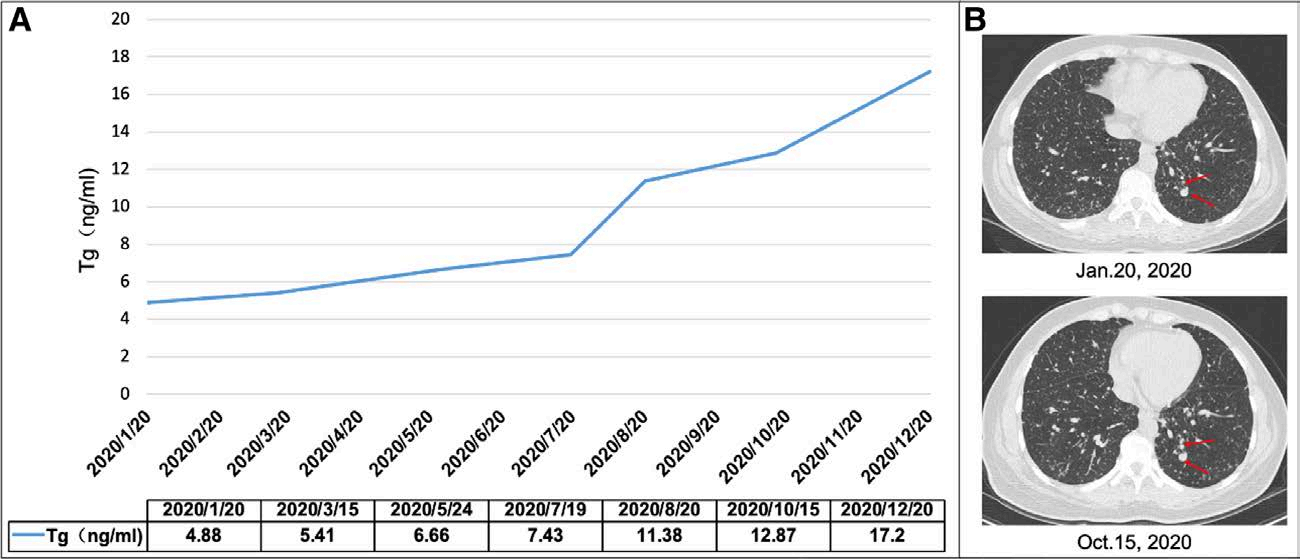
Clinical course of the patient. (A) Rises in TSH-inhibited Tg were observed after the administration of a TKI [sorafenib]. (B) Progression of lung lesions (red arrows) after the administration of a TKI [sorafenib]. TKI = tyrosine kinase inhibitor, TSH = thyroid stimulating hormone.

In the last year, the patient’s serum Tg (sTg) level and number of pulmonary lesions gradually increased, and he developed hemoptysis (Fig. 1). Because most lung lesions were <1 cm, therapeutic response was evaluated by combining Response Evaluation Criteria in Solid Tumors 1.1 criteria with changes in sTg levels. Changes in sTg levels were used to evaluate treatment response {[partial response, PR]: sTg decreased by more than 30% from baseline; [progressive disease, PD]: sTg increased by more than 25% from baseline; [SD]: change between PR and PD (−30% ~ +25%)}. His therapeutic response was evaluated as PD.

## 4. Diagnostic assessment

**Figure 2.**
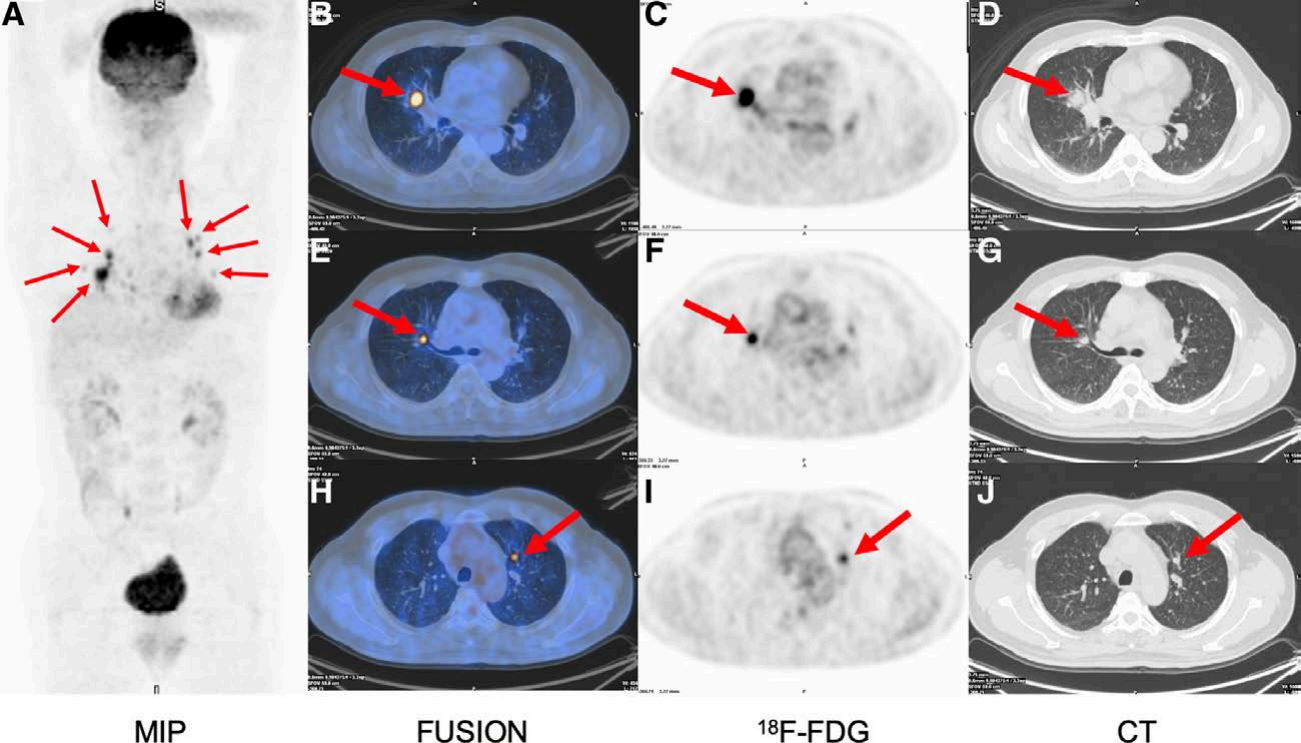
^18^F-FDG PET/CT. (A) ^18^F-FDG MIP showing multiple lung metastases with increased FDG uptake (red arrows). (B, E, H) Transaxial fused PET/CT. (C, F, I) Transaxial PET. (D, G, J) Transaxial CT. ^18^F-FDG = 2-deoxy-2-[^18^F]fluoro-D-glucose, MIP = maximum intensity projection.

**Figure 3.**
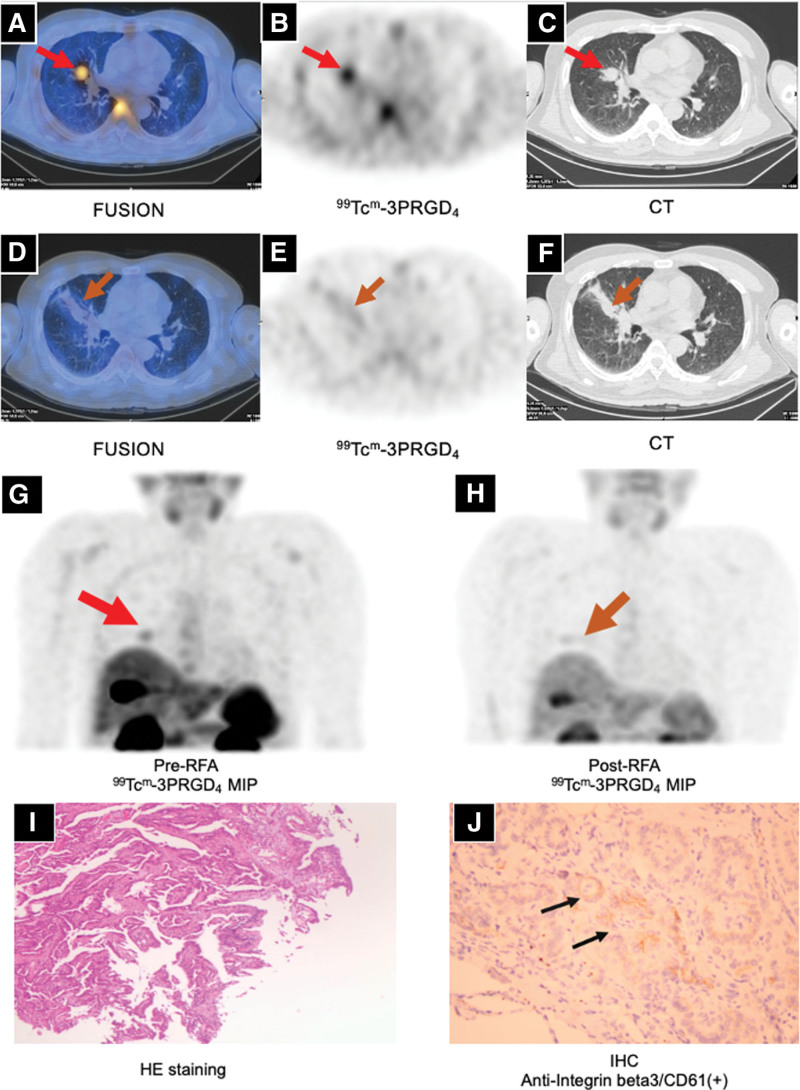
^99^Tc^m^-3PRGD_2_ SPECT/CT and IHC. (A–C, G) ^99^Tc^m^-3PRGD_2_ SPECT/CT before RFA showed only 1 lesion with increased RGD uptake in the right middle lung (red arrow). (D–F, H) ^99^Tc^m^-3PRGD_2_ SPECT/CT after RFA showed postoperative changes in the middle lobe of the right lung (brown arrow). (I) Hematoxylin-eosin staining confirmed adenocarcinoma. (J) Immunohistochemistry staining against anti-integrin α_v_β_3_ antibody showed weak positivity (black arrow). IHC = immunohistochemistry, RFA = radiofrequency ablation, RGD = arginine-glycine-aspartic, ^99^Tc^m^-3PRGD_2_ = 99technetiumm-three polyethylene glycol spacers-arginine-glycine-aspartic acid.

The sTg value with thyroid stimulating hormone inhibition was 17.2 ng/mL before PET/CT examination. He underwent both ^18^F-FDG PET/CT and ^99^Tc^m^-3PRGD_2_ SPECT/CT with an interval of 2 days. He did not receive any antineoplastic therapy between the 2 scans. The ^18^F-FDG PET/CT showed multiple lesions with increased FDG uptake in the lung. Multiple lesions with moderate to intense hypermetabolism (average standard uptake value [SUV]: 5.4, SUV_max_: 4.3–10.5, maximum diameter of lesions: 0.5–1.8 cm) were detected on the axial ^18^F-FDG lung images (Fig. 2). ^99^Tc^m^-3PRGD_2_ SPECT/CT showed only 1 lesion with arginine-glycine-aspartic (RGD) uptake. There was moderate RGD uptake (SUV_max_: 3.8, maximum diameter of lesion: 1.8 cm) in the middle lobe of the right lung (Fig. 3A–C and G). We found that the dimension doubling rate of RGD-positive lesion was significantly higher than that of RGD-negative lesion.

## 5. Therapeutic intervention

Radiofrequency ablation (RFA) was performed for only the lesion with RDG and FDG positivity. A needle biopsy of the lesion was performed prior to RFA, hematoxylin-eosin staining of the lesion with RGD uptake confirmed adenocarcinoma. Immunohistochemistry (IHC) found the following: PAX8 (++++), NaPsinA (−), Tg (+++), TTF1 (++++), and Ki67 (3%). Based on the combination of hematoxylin-eosin and IHC results, lung metastasis of TC was considered. Supplementary IHC showed weakly positive (+) staining for the anti-integrin αvβ_3_ antibody, or CD61 (Fig. 3I and J). After RFA, the sorafenib treatment regimen was maintained.

## 6. Follow-up and outcomes

**Figure 4.**
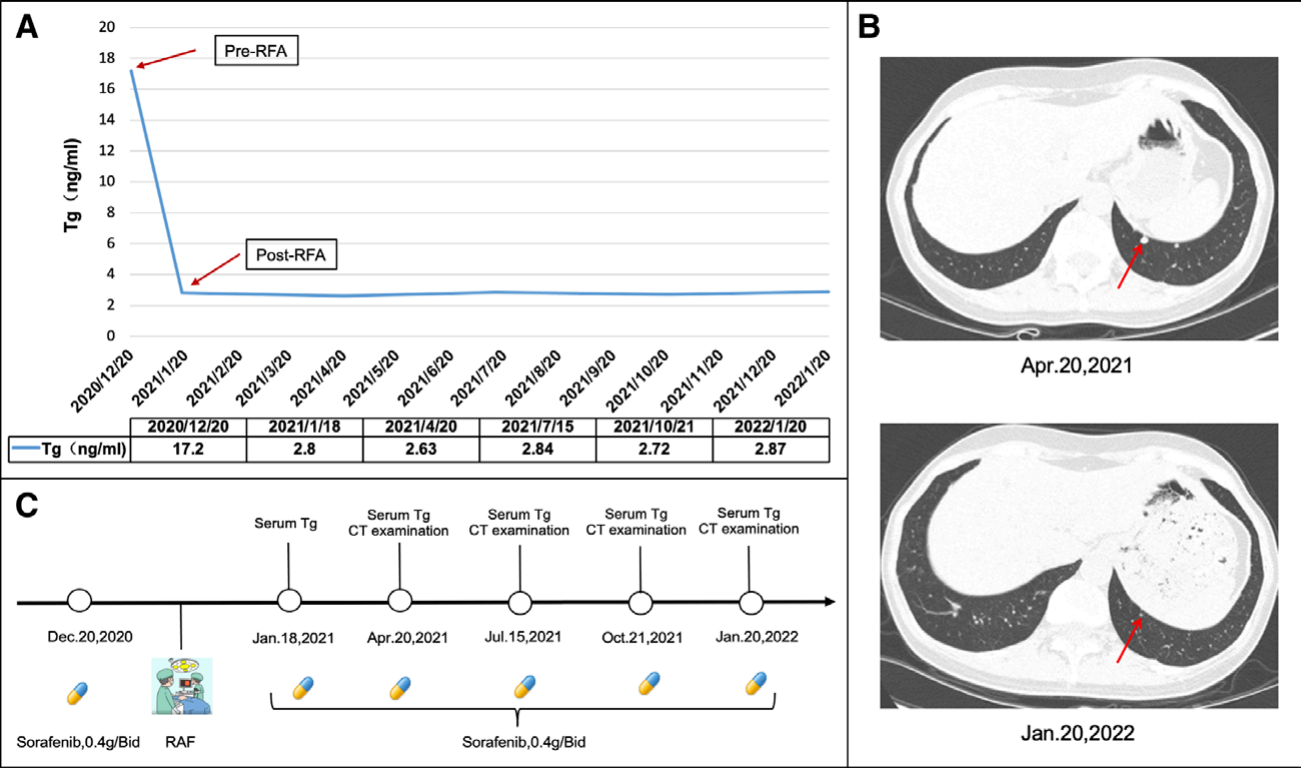
Clinical follow up of the patient. (A) TSH-inhibited Tg levels were stable after RFA. (B) At follow-up, some lung nodules were reduced in size (red arrow). (C) Timeline of the diagnostic and therapeutic process during follow-up. RFA = radiofrequency ablation, TSH = thyroid stimulating hormone.

The ^99^Tc^m^-3PRGD_2_ scan showed no RGD uptake in the right middle pulmonary lobe (Fig. 3D–F and H). His sTg level decreased by 83.7% (the Tg value was 2.8 ng/mL, and the thyroid stimulating hormone value was <0.01 mIU/L). Moreover, chest CT and sTg tests were routinely performed every 3 months to assess changes in the lung lesions, and therapeutic response was evaluated as PR at follow-up (>12 months) (Fig. 4). The patient’s hemoptysis disappeared.

## 7. Discussion

Integrin α_v_β_3_ has been found to be a key factor in tumor angiogenesis that is necessary for tumor growth. Integrin α_v_β_3_ is also important in regulating the potential of cancer cells to metastasize, influencing cell motility by interacting with fibronectin, and enhancing the survival of cancer cells in circulation by increasing their resistance to isolation-inducing cell death.^[6]^ RGD is a small peptide containing arginine, glycine and aspartic acid. RGD has a ligand‒receptor relationship with the integrin ανβ3 receptor and has high selectivity and affinity.^[7]^ The study by Zhao et al^[8]^ used ^99^Tc^m^-RGD SPECT/CT to show that the integrin receptor reflected tumor neovasculature and that angiogenesis was active in RAIR-DTC lesions. Studies have reported that the expression of vascular endothelial growth factor is higher in TC tissue than in normal thyroid tissue.^[9–11]^ These results provide a theoretical basis for the application of TKIs in RAIR-DTC. ^99^Tc^m^-3PRGD_2_ is a novel radiotracer based on RGD that targets the integrin α_v_β_3_ receptor and can be used for the localization and growth evaluation of RAIR metastases.^[8,12–14]^ Compared with ^18^F/^68^Ga-labeled tracers, ^99^Tc^m^-3PRGD_2_ is a single-photon tracer that has good physical properties, is economical, and has wide availability.

The management of RAIR-DTC metastases has always been a challenge, especially in patients with multiple metastases that cannot be completely removed surgically. The currently available options are limited.^[15,16]^ The treatment options include external beam radiation therapy, chemotherapeutic drugs such as cisplatin, doxorubicin and taxanes, and TKIs such as apatinib and sorafenib. In this case, the ^99^Tc^m^-3PRGD_2_ scan showed increased uptake as long as the lesion had weak α_v_β_3_ receptor expression, demonstrating the high sensitivity of ^99^Tc^m^-3PRGD_2_. Despite the progressive RAIR metastases, the therapeutic response was evaluated as PD after TKI treatment. Our ^18^F-FDG and ^99^Tc^m^-3PRGD_2_ dual-tracer imaging revealed the following: if the lesion is FDG positive but RGD negative, although active glycolysis may still occur in the tumor, the proliferation ability of the vascular endothelial cells in RAIR metastasis may be inhibited, and these RAIR metastases can continue to be effectively treated by maintaining the initial KTI treatment regimen; continuation of the original TKI regimen was effective in these lesions. If the lesion is both FDG and RGD positive, the current TKI treatment regimen cannot repress tumor growth, and it is essential to revise the therapeutic strategy, such as to RFA, ^125^I-seed brachytherapy, or another TKI drug. Patient prognosis might improve by prioritizing treatment for this kind of lesion.

## 8. Conclusion

Combined multimodal imaging with ^99^Tc^m^-3PRGD_2_ SPECT/CT and ^18^F-FDG PET/CT may play some role in the management of RAIR-DTC metastases and guide subsequent therapy.

## Acknowledgments

The authors thank the Medical isotopes Research Center of Peking University for supplying HYNIC-3PRGD_2_ lyophilized kit.

## Author contributions

**Conceptualization:** Yu Zhang, Wenxin Chen.

**Data curation:** Yu Zhang.

**Investigation:** Yuxuan Li, Wenxin Chen.

**Methodology:** Yuxuan Li.

**Project administration:** Wenxin Chen.

**Resources:** Zhiyi Lin, Wenxin Chen.

**Software:** Yu Zhang.

**Supervision:** Zhiyi Lin, Wenxin Chen.

**Visualization:** Zhiyi Lin.

**Writing – original draft:** Yu Zhang.

**Writing – review & editing:** Wenxin Chen.
